# Living Donor Liver Transplantation in Biliary Atresia Children with Pulmonary Hypertension

**DOI:** 10.7150/ijms.34073

**Published:** 2019-08-14

**Authors:** Xiao-Yan Meng, Mi-Yuan Chen, Zhi-Ying Pan, Ye-Feng Lu, Wei Wei, Yu-Gang Lu

**Affiliations:** 1 Department of Anesthesiology, Eastern Hepatobiliary Surgery Hospital, Second Military Medical University, Shanghai, 200433, China;; 2 Department of Anesthesiology, Shanghai Pulmonary Hospital, Tongji University School of Medicine, Shanghai, 200433, China;; 3 Department of Anesthesiology, Renji Hospital, Shanghai Jiao Tong University School of Medicine, Shanghai, 200127, China.; 4 Department of Hepatic Surgery, Renji Hospital, Shanghai Jiao Tong University School of Medicine, Shanghai, 200127, China.; 5 Department of Medical Imaging-Ultrasound, JiaHui International Hospital, Shanghai, 200233, China.

**Keywords:** pulmonary hypertension, living donor liver transplantation, pediatric liver transplantation, outcomes.

## Abstract

Objective: Though living donor liver transplantation (LDLT) is commonly performed for pediatric patients with biliary atresia (BA), pulmonary hypertension (PH) is seldom encountered or reported previously. The aim of this study is mainly to identify the prevalence of PH in pediatric patients undergoing liver transplantation and assess whether PH significantly augment the operative risk and evaluate the outcomes in this series of patients.

Design: Retrospectively cohort study.

Setting: Renji hospital, Shanghai, China.

Participants: This study comprised 161 pediatric patients undergoing LDLT.

Interventions: Patient diagnosed of PH in preoperative examination was compared to those without PH in intra- or post- operative complications or outcomes.

Measurements and Main Results: We collected clinical records of LDLT surgery for pediatric patients during the year of 2016 in our hospital. Results suggested that pediatric patients undergoing LDLT had a substantial number of PH with a prevalence of 16.1% in this study. No significant difference was identified between two groups of patients regarding intraoperative outcomes and postoperative complications and mortality.

Conclusion: LDLT is a safe procedure in a selected group of BA patients with PH, however, further long-term clinical investigations and mechanical researches are needed.

## Introduction

Living donor liver transplantation (LDLT) is commonly performed today and has become the gold standard for pediatric patients with end-stage liver disease (ESLD) 1. Biliary atresia (BA) ranks as the main cause for liver transplantation (LT) in children 2. Currently evidences indicate that direct hyperbilirubinemia and bile acid excess are directly associated with dysfunctions in the cardiovascular functions 3, 4. It is also well recognized that the chronic liver disease is associated with respiratory symptoms and hypoxia 5. There are numerous studies in recent years investigating the risks of pulmonary complications and abnormalities specific to chronic liver disease, including hepatopulmonary syndrome, portopulmonary hypertension, and hepatic hydrothorax in LT 6-8. These studies suggest that LT has established itself as an effective treatment for these selected patients. While the contraindication for LT has been discussed for many times, pulmonary hypertension (PH) is seldom encountered or reported in pediatric LT previously.

PH is a haemodynamic pathophysiological condition with an unfavorable prognosis, which is defined as a mean pulmonary artery pressure (mPAP) of ≥25 mmHg at rest and generally measured by right-heart catheterization 9. PH can be found in multiple clinical conditions, which is characterized by increasing in pulmonary arterial pressure (PAP), endothelial dysfunction and pulmonary vascular resistance. These series of pathophysiological changes may ultimately lead to a reversal of the systemic-to-pulmonary shunt accompanied by cyanosis, the so-called Eisenmenger syndrome 10. What's more, coexisting of severe liver dysfunction may also deteriorate the intrapulmonary vascular dilatation and dysfunction, as clinicians often argue that these types of patients may not tolerate LT to survive.

There is no doubt that severe PH must be corrected before LT, but pediatric patients with moderate and mild PH associated with BA pose a difficult therapeutic dilemma as cardiopulmonary anomalies are a relative contraindication to LDLT, and previous researches reported that LDLT is a comparatively safe procedure in pediatric patients with congenital heart disease 11, 12.

The aims of this study were to identify the prevalence of PH in pediatric patients undergoing LDLT, to assess whether PH significantly augment the operative risk and to evaluate the outcomes in this series of patients. We also assessed the coexistence of PH with congenital cardiovascular anomalies among BA patients. Finally, we evaluated the intraoperative hemodynamic changes and some anesthesia related complications, which present specific considerations as well as the clinical approach guiding anesthesia.

## Methods

We analyzed the medical records of all 186 pediatric patients who underwent LDLT for BA from January 1th, 2016 to January 1th, 2017. Clinical follow-up was up to June 2018. Data were analyzed retrospectively in a cohort study considering any pulmonary arterial hypertension affecting these patients. This work has been approved by Renji Hospital Ethics Committee.

Patients were enrolled in this cohort if they: (1) age under 18-year and weight less than 40 kg (pediatric patients); (2) underwent LDLT for ESLD; (3) available and complete clinical records of the patients' peri-transplantation procedure. Patients were excluded if they: (1) did not received any echocardiography or other cardiovascular investigation; (2) underwent LT for acute liver failure, metabolic disease and cancer; (3) incomplete clinical and instrumental follow-up. PH is diagnosed using continuous Doppler echocardiography, as right atrial pressure is estimated preferably from the diameter of the inferior vena cava and its inspiratory collapsibility 13, the peak tricuspid regurgitation velocity was estimate at the meantime in patients with tricuspid regurgitation, then both systolic PAP and mPAP can be calculated according to simplified form of the Bernoulli equation 14.

Echocardiography investigation was applied in 174 patients. PH was diagnosed with a mPAP≥ 25 mmHg and peak tricuspid regurgitation velocity (TRV) ≥ 2.8m/s 9, 15. Some other patients underwent echocardiography investigation and did not fulfill the PH criteria before LDLT were considered as the control cohort, being exactly homogeneous to the case cohort except for the PAP.

### Data collection and statistical analysis

For each patient the baseline characteristics were screened: sex, age, weight, height, pediatric end-stage liver disease (PELD) score, and the implement of Kasai procedure was also recorded. Moreover, we also collected donor related information, including: age, body mass index, graft weight, relationship with patients and graft-to-recipient weight ratio (GRWR).

Intraoperative outcomes were screened from the clinical files and considered as follows: blood loss, red blood cell transfusion, albumin transfusion, fluid transfusion, operation time, anhepatic phase time, cold ischemia time, the intraoperative infusion of norepinephrine and the duration of it, the levels of blood glucose, lactic acid and central venous pressure were collected at the end of the surgery.

Postoperative outcomes were recorded from the medical and follow-up records and considered as follows: the stay time in ICU, length of stay in hospital, complications, severe complications and mortality rate at follow-up period. Complications were classified according to the Clavien-Dindo's classification of surgical complications 16, and gradeⅣ and grade Ⅴ were identified as severe complications.

Data was described using descriptive statistics (mean/standard deviation or median/range for continuous variables; absolute number/frequency distribution for categorical variables). To adjust confounding between cohorts, we did Chi-squared test and Student t-test or Wilcoxon-Mann-Whitney test for categorical variables and continuous variables as appropriate. The significance level was set at P<0.05. All statistical analyses were performed using SPSS (version 25.0, College Station, TX).

## Results

186 pediatric LDLT were performed in the year of 2016. Amongst them, 25 patients were excluded from this study, 13 of them were diagnosed for other diseases other than BA, 8 of them were excluded because of incomplete clinical data or insufficient cardiovascular investigation test, two of them were without follow-up records, and two patients of them had acute liver failure. Among the 161 patients fulfilling the inclusion criteria, 26 of them were diagnosed of PH, with a prevalence rate of 16.1%, and only one (0.6%) patient hada PAP of more than 40 mmHg.

Demographic characteristics of the recipients with and without PH as well as their donors are presented in **Table 1**. The p values calculated for these variables show that the two groups are comparable ruling out the possibility of these parameters being confounders (p > 0.05). It is noteworthy that pediatric patients underwent LDLT were mainly because of BA in this research, with a median (IQR) age of 7 (6, 9) months, a mean (SD) body weight of 7.6 (2.74) kg, a mean (SD) donor age of 30.5 (6.98) years, and donors were mainly the recipients' parents.

Preoperative echocardiography revealed that 41 (25.4%) recipients had congenital cardiovascular anomalies in this cohort. **Table 2** showed the types of congenital cardiovascular anomalies and the cases of each type. Among them, atrial septal defect was the most common one, with 24 (17.8%) cases in non-PH group and 8 (28.6%) cases in PH group, accounting for 19.8% of all cases. However, no significant difference was found between the prevalence of cardiovascular anomalies in PH group and non-PH group (32.1% vs. 23.7%, P = 0.320).

The intraoperative outcomes were shown in **Table 3**. We analyzed the amount of blood loss, red blood cell transfusion, albumin transfusion, the infusion of norepinephrine as well as its' infusion duration, the operation time, the anhepatic phase and cold ischemia time, we also studied the levels of blood glucose, lactic acid and central venous pressure of the recipients at the end of procedure. The results showed that no difference exist in these intraoperative parameters between the two groups.

The postoperative complications as well as outcomes were shown in **Table 4**. No significant difference was detected for complications or severe complications in the two groups. Mortality rateiwas three patients out of 26 in the PH group (11.5%) and 11 patients out of 135 in the control group (8.1%), no statistical significance was found in this study for mortality (P=0.464). ICU stay time after the procedure was comparable in the two groups. Length of stay after surgery was the only significant result in this study (p = 0.032, student t test). Patients with PH stayed longer in hospital (32.7 ± 34.65 days) compared with the patients from the control group (25.1 ± 16.15 days), however, this difference was not significant when using non-parameter test.

## Discussion

The association of BA and cardiopulmonary disease has often been described as syndromic^17^. The current literatures scarcely focus on pediatric patients with BA and PH, as most of the recent researches consist of comorbidity of BA with congenital heart disease. Likewise, the management of PH patients undergoing LDLT is often left to the individual choice of anesthesiologists with limited experience due to the paucity of cases they encountered. While mPAP > 50 mmHg is generally considered as an absolute contraindication to LT 18, 19. Dr. Houlihan et al. 20 have concluded in a review study that LT was an effective therapy for patients with hepatopulmonary syndrome and portopulmonary hypertension, however, rigorous screening and early identification of these conditions are needed sorely. Nevertheless, it remains elusive whether the mild and moderate pulmonary arterial hypertension that do not necessarily need a prompt correction but possibly highly increase the operative risk could affect outcomes or prognoses of patients and procedures. What's more, patients with PH undergoing the procedure of LT have never been compared with those patients without PH. Our study compared two cohorts of patients in order to evaluate if any difference in complications and outcomes perioperatively.

Our results suggested that the prevalence of PH in patients undergoing LDLT as high as 16.1% in the year of 2016 in a single center. We demonstrated for the first time that pediatric patients undergoing LDLT had a substantial number of pulmonary arterial dysfunction at the time of surgery, and most of them were even unknown or undetected because of their benign nature. Among these patients diagnosed of PH, only 1 (3.6%) of them with mPAP over 40 mmHg, as 18 (64.2%) of them ranging from 25 mmHg to 35 mmHg, which is recognized as mild PH. Due to the characteristics of the data and the diagnose criteria, this study did not conclude the harms of PH on LDLT in children, but provided anesthesiologists and clinicians with an overview about the risks of easily neglected moderate and mild PH in LDLT. Moreover, as the relationship between congenital heart disease with both BA and PAH has been reported previously, we also identified that the prevalence of congenital cardiovascular anomalies in PH group is not higher compared to Non-PH group, it implied that there have no significant association between PH and congenital cardiovascular anomalies in BA patients according to this study. However, BA related hypoalbuminemia, massive ascites and jaundice could induce could also be deteriorated in co-existence of pulmonary congestion and cardiovascular decompensation 21. It is crucial to identify which condition caused the patient's symptoms that need immediate intervention before transplantation.

The most important results from our series of 26 patients with PH were the intra-operative and postoperative complications. Our results identified no significant difference between the two groups of patients, regarding blood loss, RBC transfusion, ALB transfusion, fluid transfusion, operation time, the intraoperative infusion of norepinephrine and its duration, and the level of blood glucose, lactic acid and central venous pressure at the end of surgery. These similar pattern of parameters between the two cohorts indicated that anesthesiologists do not face greater difficulties or unexpected events perioperatively.

Further, no difference was observed in the postoperative complications or severe complications which need medication or intervention. These results indicated that mild or moderate PH diagnosed of echocardiography do not significantly influence the operative risk of these patients. The only one statistically significant result we obtained was that children with PH do stay longer in hospital (32.4 ± 34.75 days) compared with the control group (23.6 ± 15.25 days), however this difference was not that significant when analyzed in non-parameter test considering the distribution of data. Further studies with larger samples are need to evaluate the potential difference in hospital stay time. Mortality is comparable in the two groups during the follow-up time. The PH group showed a mortality rate of 10.7% with their counterparts arrive at 7.5%. Considering small number of cases, further studies with larger samples and longer follow-up are helpful to infer more accurate conclusion on it.

Our results in the present study demonstrated that LDLT can be safely performed in pediatric patients with PH as long as the indications for LT are clear. It remains an interesting question that whether the existence of PH in these children is a complication of liver disease, which might be well reversible after transplantation surgery or it's an independent disease which is magnified by pre-existing liver dysfunction and could affect the long-term life quality for these patients. Answering these questions needs both long-term clinical observation and animal research. Recently researches have hypothesized that significant portal shunting pre- and post-operation could expose the pulmonary vascular bed to additional shear stress and vasoactive mediators, thus contribute to pulmonary arterial vasoconstriction and remodeling 22. What's more, studies on idiopathic pulmonary arterial hypertension (IPAH) have already suggested that the pathology of IPAH is triggered by vascular injury reflected by the development of plexiform arteriopathy, concentric intimal fibrosis, and proliferation and muscularization of the pulmonary arterioles 23, it also relates to germline mutations as well as genetic alterations in serotonin transport 24, 25. Whether PH in children with ESLD presenting similar pathological changes needs further investigation.

Notably, several methodological discrepancies or limitations should be taken into consideration. Firstly, considering the very nature of the retrospective cohort study, the parameters and information we analyzed are all based on the completeness of the medical records, with some biases and inaccuracies might exist. Meanwhile, several important parameters were not included because of insufficient clinical data or did not perform the investigation before and after surgery. For instance, perioperative hypoxemia and blood pressure change were not recorded. Echocardiography was not performed neither after surgery nor in postoperative re-examination, thus, we did not investigate whether the process of LDLT could influence circulatory and respiratory function in pediatric patients with PH. Secondly, according to the most recent European Society of Cardiology and European Respiratory Society (ESC/ERS) Guidelines for the diagnosis and treatment of PH in 2015, mPAP ≥ 25 mmHg using right-heart catheterization is considered as golden standard for diagnosis, while conclusions derived from an echocardiographic examination are better used to grade the probability of PH based on TRV at rest as well as the presence of additional pre-specified echocardiographic variables suggestive of PH 9. However, as the right-heart catheterization is rarely used in pediatrics and infants, the diagnosed PH based on echocardiographic examination in our study could comparatively provide an overview on those patients with high probability of PH which need further confirmation with invasive investigation another disadvantage is that we only screened out patients with tricuspid regurgitation, which may cause diagnose omission.

## Conclusion

In this study, we retrospectively analyzed 161 pediatric patients with ESLD undergoing LDLT. By comparing surgical outcomes and complications of those patients with or without PH, we deemed that LDLT is a safe procedure in a selected group of BA patients with PH, however, further long-term clinical investigations and mechanical researches are needed.

## Figures and Tables

**Table 1:** Baseline Characters for Recipients and Donors.

	Total (N=161)	Non-PH (N=135)	PH (N=26)	P Value
**Recipient**				
Age (month), Mean (SD)	9.89(11.57)	10.1(12.33)	8.6(5.52)	0.435
Median (IQR)	7(6,9)	7(6,9)	7(6,9)	0.548
Height (cm), Mean (SD)	67.7(9.98)	68.0(10.62)	65.8(4.98)	0.224
Weight (kg), Mean (SD)	7.6(2.74)	7.7(2.92)	7.2(1.47)	0.378
PELD, Mean (SD)	18.5(9.32)	18.3(9.67)	20.0(7.24)	0.283
Sex, Male, number (%)	69(42.8)	57(42.2)	12(46.1)	0.667
Kasai procedure, number (%)				0.983
No	63(39.1)	53(39.3)	10(38.4)	
Yes	98(60.9)	82(60.7)	16(61.5)	
**Donor**				
Age (year), Mean (SD)	30.5(6.98)	30.6(7.37)	29.5(4.68)	0.066
BMI, Mean (SD)	21.7(2.74)	21.9(2.78)	21.9(2.51)	0.436
Graft Weight (g), Mean (SD)	256.9(63.23)	252.9(57.74)	239.8(42.89)	0.460
Relationship, number (%)				0.259
Father	55(34.1)	48(35.5)	7(26.9)	
Mother	99(61.5)	80(59.2)	19(73.1)	
Others	7(4.3)	7(5.3)	0	
GRWR, number (%)				0.622
≥4%	36(22.3)	29(21.5)	7(26.9)	
<4%	125(77.6)	106(78.5)	19(73.1)	

PH, pulmonary hypertension; PELD, pediatric end-stage liver disease score; BMI, body mass index; GRWR, graft-to-recipient weight ratio.

**Table 2:** Types of Congenital Cardiovascular Anomalies

	Total (N=161)	Non-PH (N=135)	PH (N=26)	P value
Total, number (%)	41(25.4)	32(23.7)	9(32.1)	0.320
Pulmonary stenosis, number (%)	1(0.6)	1(0.7)	0	
Ventricular septal defect, number (%)	3(1.8)	2(1.4)	1(3.6)	
Patent ductus arteriosus, number (%)	1(0.6)	1(0.7)	0	
Atrioventricualr septal defect, number (%)	2(1.2)	2(1.4)	0	
Atrial septal defect, number (%)	32(19.8)	24(17.8)	8(28.6)	
Oval fossa defect, number (%)	2(1.2)	2(1.4)	0	

PH = Pulmonary hypertension.

**Table 3:** Intraoperative Outcomes

	Non-PH (N=135)	PH (N=26)	*P* Value
Blood loss (ml), Mean (SD)	175.1(124.79)	146.1(64.68)	0.345
RBC transfusion (ml), Mean (SD)	269.3(174.25)	257.8(120.44)	0.345
ALB infusion (g), Mean (SD)	23.1(8.83)	21.7(8.19)	0.451
Fluid infusion (ml), Mean (SD)	1768.9(581.9)	1704.6(426.24)	0.630
NE infusion, number (%)	21(14.4)	4(14.2)	0.989
NE infusion time (min), Mean (SD)	242.7(130.94)	229.2(154.93)	0.856
Operation time (min), Mean (SD)	396.5(75.32)	387.7(56.84)	0.559
Anhepatic phase (min), Mean (SD)	39.5(13.87)	41.2(9.85)	0.529
Cold ischemia time (min), Mean (SD)	70.2(21.67)	64.0(16.94)	0.271
Blood glucose (mmol/L), Mean (SD)	6.7(1.96)	6.8(1.48)	0.728
Lactic acid (mmol/L), Mean (SD)	1.6(1.26)	2.0(2.31)	0.288
CVP (cm H2O), Mean (SD)	8.6(2.83)	8.7(2.89)	0.938

PH, pulmonary hypertension; RBC = red blood cell; ALB, albumin; NE, norepinephrine; CVP, central venous pressure.

**Table 4:** Postoperative Outcomes

	Non-PH (N=135)	PH (N=26)	P Value
Dichotomous Outcomes			
Complications, number (%)	54(40)	7(26.9)	0.229
Severe complications, number (%)	38(28.1)	3(11.5)	0.289
Mortality, number (%)	10(8.1)	3(11.5)	0.464
Graft survivals, rate (95%CI)	0.919 (0.895, 0.943)	0.880 (0.815, 0.945)	-
Continuous Outcomes			
Extubating time, min, mean (sd.)	15.7(5.67)	18.4(8.32)	0.276
ICU stay time (days), Mean (SD)	5.2(2.72)	5.2(1.93)	0.900
Length of stay(days), Mean (SD)	25.1(16.15)	32.7(34.65)	0.032
Median (IQR)	20(17.26)	23(18, 26.25)	0.090

PH, pulmonary hypertension; IQR, inter quartile range.
